# Safety of eribulin: A real-world study based on a pharmacovigilance database

**DOI:** 10.1371/journal.pone.0356800

**Published:** 2026-09-17

**Authors:** Yao Zhou, Jie Gong, Lele Shen, Jie Ling, Shiting Wu, Anqi Ge, Lin Qi, Lifang Liu

**Affiliations:** 1 The Second Affiliated Hospital of Hunan University of Chinese Medicine, Changsha, Hunan, China; 2 The First Affiliated Hospital of Hunan University of Chinese Medicine, Changsha, Hunan, China; 3 Hunan Academy of Chinese Medicine, Changsha, Hunan, China; Peking University First Hospital, CHINA

## Abstract

Eribulin is used to treat metastatic breast cancer, but its adverse event (AE) profile requires comprehensive analysis using FDA Adverse Event Reporting System (FAERS) data to guide clinical use. We analyzed eribulin-related AE reports in FAERS (Q1 2011 - Q2 2024). Disproportionality analyses (ROR, PRR, MGPS, BCPNN) identified significant AE signals. Our study finalized 3570 AE reports with eribulin as the primary suspect drug from the FAERS database, including 7455 coded AE records. The included patients were predominantly female (90.1%) and aged 35–59 years (42.61%). Eribulin-associated AEs spanned 26 organ systems. Only “Blood and lymphatic system disorders” (n = 1,788) met significance across all four algorithms. At the preferred term level, 95 positive disproportionality signals emerged. Common expected AEs included neutropenia, febrile neutropenia, and leukopenia. Critically, serious unexpected AEs not consistently referenced in drug inserts were identified, such as pleural effusion, respiratory failure, and cardiac failure. Clinical use of eribulin requires great attention to blood and lymphatic adverse events, as well as enhanced monitoring and reporting of adverse events not mentioned in the insert to prevent serious AEs.

## Introduction

Breast cancer (BC), the most common malignant tumor in women, is estimated to have 2.3 million new breast cancer cases in 2022 alone [1]. In recent years, a variety of multimodal therapies and drugs for BC have been developed and applied, which increases the chance of cure for 70–80% of BC patients [2]. However, for patients with advanced or metastatic breast cancer (mBC), the highly aggressive and malignant nature of the disease still leads to a poor prognosis. Therefore, exploring more effective and safe treatments to improve the survival of mBC patients has become an urgent need in the field of breast cancer research.

Eribulin mesylate (Eribulin), a novel microtubule inhibitor, was approved by the U.S. Food and Drug Administration (FDA) on November 15, 2010, for treating patients with metastatic breast cancer who have previously received at least two chemotherapeutic regimens for metastatic disease [3], including an anthracycline and a taxane in the adjuvant or metastatic setting. Unlike other microtubule inhibitors, eribulin directly binds to microtubule terminals, inhibiting microtubule protein growth and causing G2/M phase cell cycle arrest [4], thus exerting its cytotoxic effects. In addition, eribulin reprograms the tumor microenvironment to promote immune cell infiltration and activation, enhancing anti-tumor immunity [5,6]. This dual action not only strengthens its own anti-tumor activity but also supports synergistic combinations with immunotherapeutic drugs [7]. Thus, the application of eribulin in the treatment of mBC is promising.

Although targeted therapies and antibody-drug conjugates have expanded, chemotherapy remains a cornerstone for many patients with mBC. Since the EMBRACE study established eribulin’s therapeutic role in mBC patients [8], several studies have confirmed the benefit of eribulin in triple-negative mBC patients, HER2 + , and HR + /HER2- mBC patients [9–13]. Clinical studies have shown that the use of eribulin causes adverse effects, such as neutropenia, fatigue, alopecia, nausea, and anemia [14], and that peripheral neuropathy, despite its low incidence [15], is often a key factor leading to treatment interruption [8]. Given the expanding clinical use of eribulin, a better understanding of its rare adverse effects is particularly important.

However, the current understanding of adverse reactions associated with eribulin relies on limited real-world clinical data, which often lack systematic, in-depth analyses, making it particularly challenging to identify rare and serious adverse events. In contrast, the U.S. FDA Adverse Event Reporting System (FAERS) serves as a publicly available, and multisource pharmacovigilance database designed to enhance postmarketing safety monitoring of drugs [16]. This study aimed to comprehensively collect and analyze eribulin-related adverse reaction reports from FAERS, identify real-world pharmacovigilance risk signals based on disproportionality analysis, and provide scientific evidence to guide the safe clinical use of eribulin.

## Materials and methods

### Data sources

Based on the FAERS database (https://www.fda.gov/drugs/drug-approvals-and-databases/fda-adverse-event-reporting-system-faers), we conducted a comprehensive analysis of AEs related to eribulin. FAERS is a publicly available database managed by the FDA that includes over 27 million adverse event reports, medication error reports, and drug quality reports [17].

We downloaded adverse event data relating to eribulin (“Eribulin,” “Eribulin mesylate,” “Eribulin mesilate”, “Halaven”) from the FAERS database website for the period from 1 January 2011 to 30 June 2024. In order to increase the credibility of the results, only data labeled as “Principal Suspect (ROLE_COD = PS)” in the drug file were selected for our study. Subsequently, according to the recommendations of the official FDA website, we used R software (Version 4.4.1) to organize the data to remove duplicates by selecting the most recent FDA_DT report when CASEID was the same, and selecting the higher PRIMARYID report when FDA_DT and CASEID were the same [18]. Ultimately, we standardized adverse events based on the Preferred Term (PT) and System Organ Class (SOC) of the Medical Dictionary for Regulatory Activities (MedDRA, version 26.1).

This study was conducted using publicly available, de-identified data from the FAERS database. As no identifiable personal information was accessed and no direct patient involvement occurred, ethical approval was not required.

### Statistical methods

**Fig 1:**
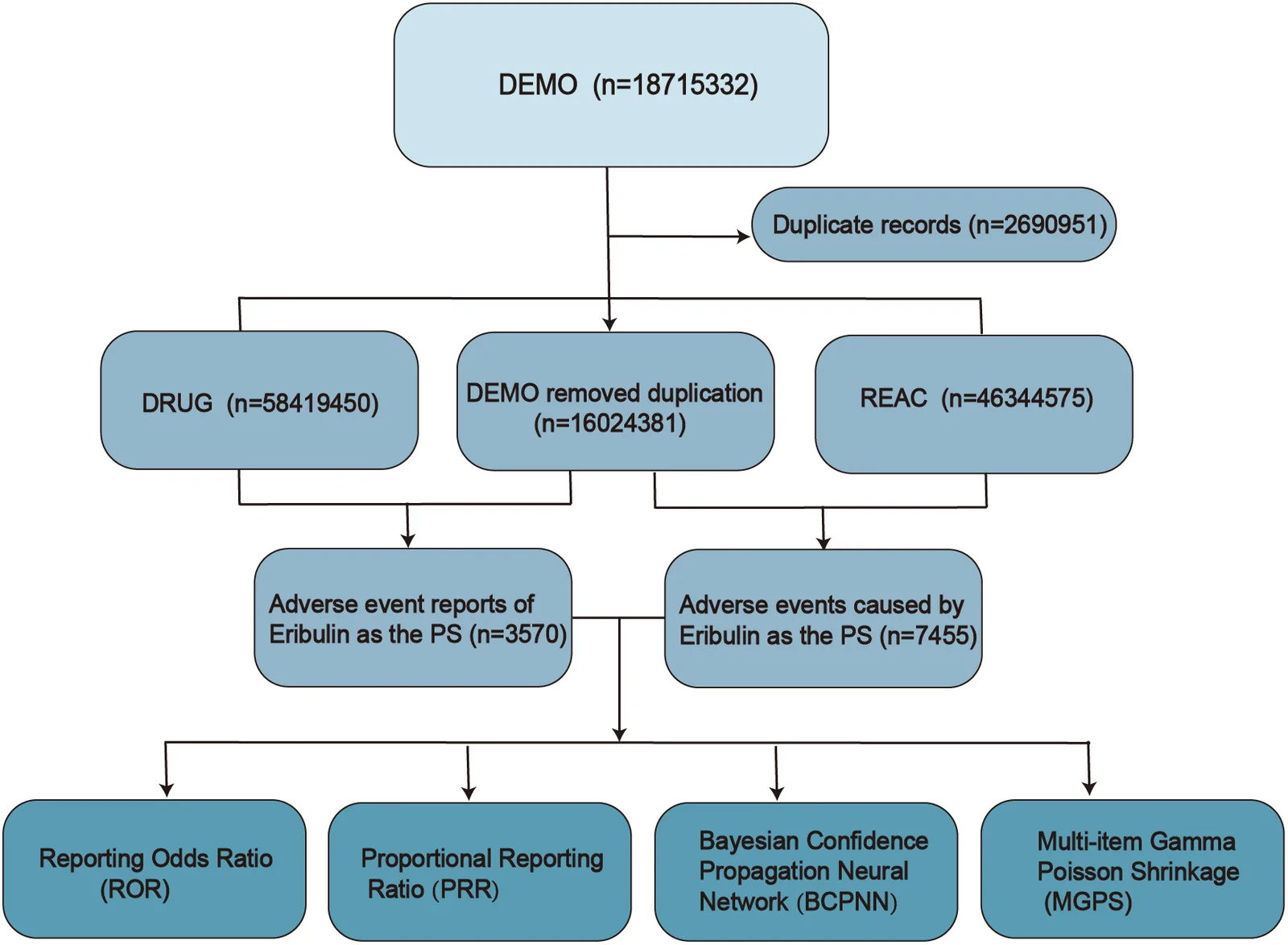
Research flow chart for this study. Note: DEMO: demographic and administrative information; DRUG: drug details; REAC: coded adverse events; PS: primary suspect.

In this study, in order to assess the association between eribulin and its adverse events, we applied disproportionality analysis to detect safety signals. Disproportionality analysis includes the reporting odds ratio (ROR), proportional reporting ratio (PRR), bayesian confidence propagation neural network (BCPNN) and the multi-item gamma poisson shrinker (MGPS) four algorithms. The calculation principles of these four algorithms are based on the 2 × 2 contingency table (S1 Table). An adverse event was defined a priori as a positive disproportionality signal only when it met the positivity criteria for all four algorithms. The formulas and positivity criteria are provided in S2 Table, and the data-analysis workflow is shown in Fig 1.

## Results

### Baseline situation statistics

Between the first quarter of 2011 and the second quarter of 2024, a comprehensive retrieval of the FAERS database yielded a total of 18,715,332 individual demographic records (DEMO). Following data deduplication and organization, we obtained 3,570 adverse event reports with eribulin as the primary suspect drug, including a total of 7,455 coded adverse reaction records (Fig 1). Further statistical analysis revealed that the number of female patients using eribulin was higher than that of male patients (90.14% vs. 7.62%) (Table 1). In terms of age, the most affected patients were those aged 35–59 years, accounting for 42.61% of the total, followed by patients over 60 years old, comprising 36.95% of the total. Among the reporters, physicians submitted the highest number of cases (n = 2,257, 63.22%), with health professionals being the second largest group (n = 585, 16.39%). Among the reporting countries, Japan (n = 1,137, 31.85%), China (n = 546, 15.29%), and the United States (n = 518, 14.51%) reported the highest number of cases.

**Table 1:** The clinical characteristics of reports of eribulin in the FAERS database (Q1 2011 to Q2 2024).

Characteristic	Reports number (N)	Proportion (%)
Overall reports	3570	
Gender		
Female	3218	90.14%
Male	272	7.62%
Missing	80	2.24%
Age (year)		
<18	17	0.48%
18-34	71	1.99%
35-59	1521	42.61%
≥60	1319	36.95%
Missing	642	17.98%
Reporter		
Physician	2257	63.22%
Health Professional	585	16.39%
Other	283	7.93%
Pharmacist	210	5.88%
Consumer	160	4.48%
Missing	70	1.96%
Registered Nurse	5	0.14%
Top 7 Reporting Countries		
Japan	1137	31.85%
China	546	15.29%
United States	518	14.51%
Italy	194	5.43%
Germany	181	5.07%
France	160	4.48%
United Kingdom	115	3.22%

**Fig 2:**
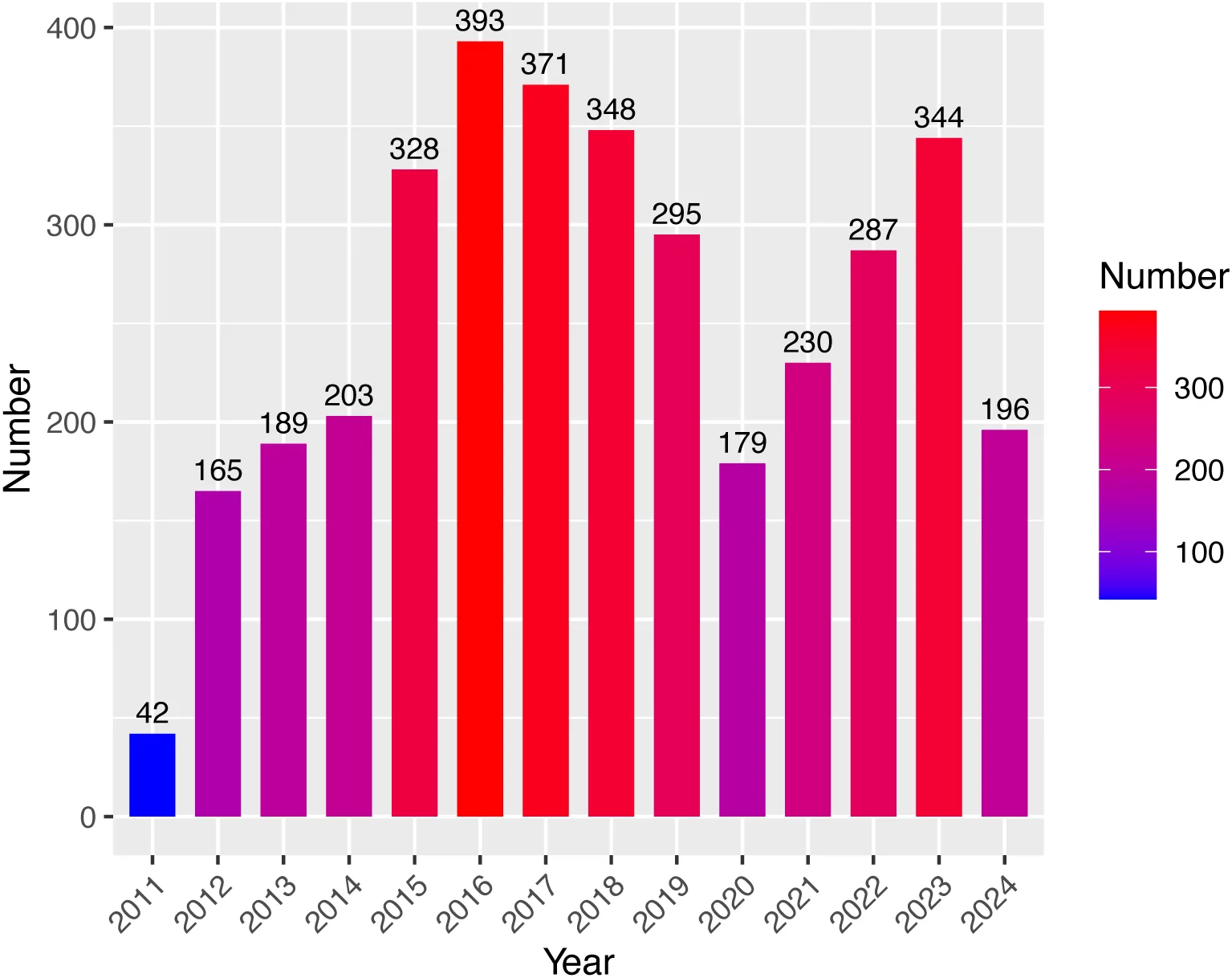
Statistics on the annual frequency of reported adverse events associated with eribulin. The decline during 2016–2020 may be related to changes in drug utilization or market dynamics (e.g., introduction of new competing therapies).

Furthermore, we summarized the annual number of AE reports related to eribulin since 2011 (Fig 2). As illustrated in Fig 2, the number of eribulin-related AE reports increased annually from 2011 to 2016, declined from 2016 to 2020, and then rose again thereafter. The initial increase (2011–2016) may reflect postmarketing accumulation of clinical use and increased reporting awareness; the subsequent decline (2016–2020) may relate to changes in drug usage or market dynamics; the subsequent rise (post 2020) may be linked to new clinical concerns or safety monitoring updates.

### Signal detection based on system organ classes

The initial search identified 7,455 coded adverse reaction records. After excluding 20 records where both the PT and SOC fields were blank, the remaining 7,435 coded reaction records were included in the SOC level analysis of adverse reactions.

**Fig 3:**
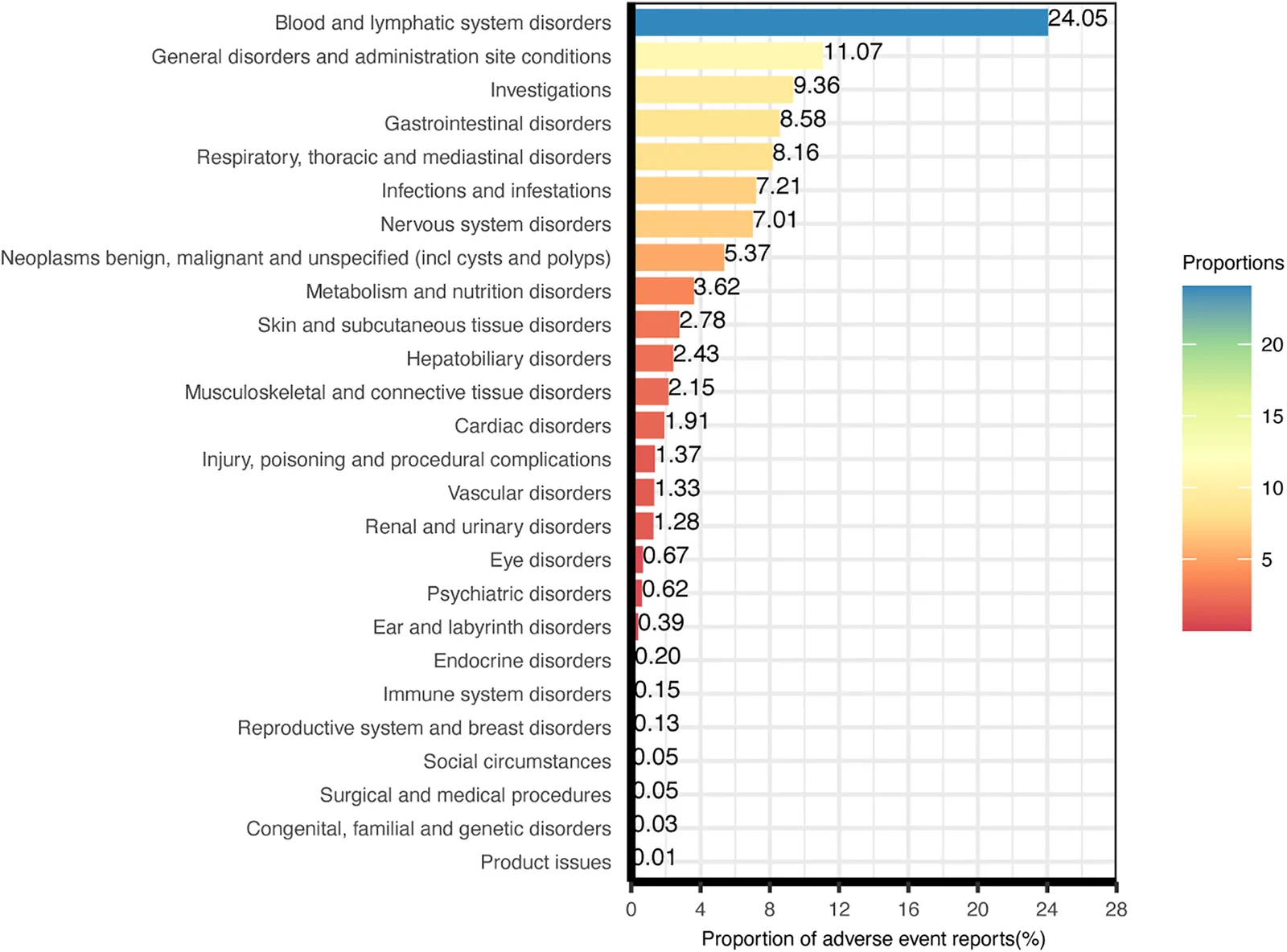
26 System Organ Classes(SOCs) associated with eribulin, with labeled percentage values indicating the proportion of eribulin-related adverse event reports for each SOC.

A total of 26 different system organ classes (SOCs) were involved in the reports associated with eribulin (Table 2, Fig 3). Based on disproportionality analysis at the SOC level, only blood and lymphatic system disorders (n = 1788) simultaneously met all four algorithms: ROR, PRR, BCPNN, and MGPS. Hepatobiliary disorders (n = 181) met three algorithms (ROR, PRR, and MGPS). The following SOCs fulfilled only the ROR algorithm: Investigations (n = 696), Respiratory, thoracic and mediastinal disorders (n = 607), Infections and infestations (n = 536), Neoplasms benign, malignant and unspecified (incl cysts and polyps) (n = 399), and Metabolism and nutrition disorders (n = 269) (Table 2).

**Table 2:** SOC-level disproportionality signals for eribulin.

System Organ Class (SOC)	Case numbers	ROR(95%CI)	PRR (χ2)	EBGM(EBGM05)	IC(IC025)
Blood and lymphatic system disorders	1788	18.74 (17.76 - 19.76)*	14.48 (22766.86)*	14.45 (13.82)*	3.85 (2.19)*
General disorders and administration site conditions	823	0.57 (0.53 - 0.61)	0.62 (236.8)	0.62 (0.58)	−0.69 (−2.36)
Investigations	696	1.65 (1.53 - 1.79)*	1.59 (163.32)	1.59 (1.49)	0.67 (−0.99)
Gastrointestinal disorders	638	1.01 (0.93 - 1.1)	1.01 (0.06)	1.01 (0.94)	0.01 (−1.65)
Respiratory, thoracic and mediastinal disorders	607	1.78 (1.64 - 1.93)*	1.72 (190.78)	1.72 (1.6)	0.78 (−0.89)
Infections and infestations	536	1.36 (1.25 - 1.49)*	1.34 (48.1)	1.34 (1.24)	0.42 (−1.25)
Nervous system disorders	521	0.83 (0.76 - 0.91)	0.84 (16.34)	0.84 (0.78)	−0.24 (−1.91)
Neoplasms benign, malignant and unspecified (incl cysts and polyps)	399	1.97 (1.78 - 2.18)*	1.92 (180.7)	1.92 (1.76)	0.94 (−0.73)
Metabolism and nutrition disorders	269	1.75 (1.55 - 1.98)*	1.73 (84.16)	1.73 (1.56)	0.79 (−0.88)
Skin and subcutaneous tissue disorders	207	0.49 (0.43 - 0.56)	0.51 (105.79)	0.51 (0.45)	−0.98 (−2.65)
Hepatobiliary disorders	181	2.84 (2.45 - 3.29)*	2.79 (209.7)*	2.79 (2.47)*	1.48 (−0.19)
Musculoskeletal and connective tissue disorders	160	0.39 (0.33 - 0.45)	0.4 (151.52)	0.4 (0.35)	−1.32 (−2.99)
Cardiac disorders	142	0.79 (0.67 - 0.93)	0.79 (7.91)	0.79 (0.69)	−0.34 (−2)
Injury, poisoning and procedural complications	102	0.13 (0.1 - 0.15)	0.14 (614.9)	0.14 (0.12)	−2.87 (−4.53)
Vascular disorders	99	0.63 (0.52 - 0.77)	0.64 (20.52)	0.64 (0.54)	−0.64 (−2.31)
Renal and urinary disorders	95	0.68 (0.55 - 0.83)	0.68 (14.59)	0.68 (0.57)	−0.56 (−2.22)
Eye disorders	50	0.33 (0.25 - 0.44)	0.34 (66.69)	0.34 (0.27)	−1.57 (−3.24)
Psychiatric disorders	46	0.11 (0.08 - 0.14)	0.11 (345.32)	0.11 (0.09)	−3.17 (−4.83)
Ear and labyrinth disorders	29	0.9 (0.63 - 1.3)	0.9 (0.32)	0.9 (0.66)	−0.15 (−1.82)
Endocrine disorders	15	0.79 (0.47 - 1.31)	0.79 (0.87)	0.79 (0.51)	−0.35 (−2.01)
Immune system disorders	11	0.13 (0.07 - 0.23)	0.13 (65.55)	0.13 (0.08)	−2.96 (−4.62)
Reproductive system and breast disorders	10	0.16 (0.09 - 0.3)	0.16 (42.86)	0.16 (0.1)	−2.61 (−4.27)
Social circumstances	4	0.13 (0.05 - 0.34)	0.13 (24.2)	0.13 (0.06)	−2.98 (−4.65)
Surgical and medical procedures	4	0.04 (0.01 - 0.1)	0.04 (95.84)	0.04 (0.02)	−4.68 (−6.34)
Congenital, familial and genetic disorders	2	0.09 (0.02 - 0.36)	0.09 (18.5)	0.09 (0.03)	−3.48 (−5.14)
Product issues	1	0.01 (0 - 0.05)	0.01 (127.79)	0.01 (0)	−7 (−8.66)

Note: * Stands for positive signals. ROR: Reporting Odds Ratio; PRR: Proportional Reporting Ratio; EBGM: Empirical Bayes Geometric Mean; IC: Information Component; IC025, Lower 2.5th percentile of the information component.

In terms of the frequency of reports for each SOC (Fig 3), the top 5 SOCs, based on the number of reports, were as follows: blood and lymphatic system disorders (n = 1788, 24.05%), general disorders and administration site conditions (n = 823, 11.07%), investigations (n = 696, 9.36%), gastrointestinal disorders (n = 638, 8.58%), and respiratory, thoracic, and mediastinal disorders (n = 607, 8.16%).

### Signal detection based on preferred terms

At the preferred term (PT) level, we used four algorithms to analyze adverse event reports, and the results showed a total of 95 positive disproportionality signals related to eribulin that satisfied the four algorithms, involving 15 SOCs. In Table 3, we show the top 30 PTs and their corresponding SOCs in descending order of the number of reports among the 95 positive disproportionality signals. According to Table 3, we can see that neutropenia (n = 556), febrile neutropenia (n = 413), malignant neoplasm progression (n = 299), myelosuppression (n = 291), and leukopenia (n = 195) were the most common signals associated with eribulin, and the neurological system showed neuropathy peripheral (n = 131). The top 10 reported adverse events were consistent with the drug insert except for “malignant neoplasm progression”.

**Table 3:** Top 30 PTs and their corresponding SOCs in descending order of number of reports among positive adverse events.

Preferred Term (PT)	System Organ Class (SOC)	Number of reports	ROR (95% CI)	PRR (χ2)	EBGM (EBGM05)	IC (IC025)
Neutropenia	Blood and lymphatic system disorders	556	36.04 (33.05 - 39.3)	33.42 (17434.22)	33.25 (30.93)	5.06 (3.39)
Febrile neutropenia	Blood and lymphatic system disorders	413	55.73 (50.44 - 61.56)	52.69 (20790.36)	52.26 (48.08)	5.71 (4.04)
Malignant neoplasm progression	Neoplasms benign, malignant and unspecified (Incl cysts and polyps)	299	25.02 (22.28 - 28.09)	24.05 (6592.04)	23.96 (21.75)	4.58 (2.92)
Myelosuppression	Blood and lymphatic system disorders	291	109.68 (97.45 - 123.43)	105.43 (29611.3)	103.69 (93.93)	6.7 (5.03)
Leukopenia	Blood and lymphatic system disorders	195	34.47 (29.89 - 39.75)	33.59 (6137.75)	33.42 (29.66)	5.06 (3.4)
Neutrophil count decreased	Investigations	188	39.36 (34.04 - 45.51)	38.39 (6808.77)	38.16 (33.79)	5.25 (3.59)
Pyrexia	General disorders and administration site conditions	183	4.48 (3.87 - 5.19)	4.39 (481.81)	4.39 (3.88)	2.13 (0.47)
White blood cell count decreased	Investigations	177	13.44 (11.58 - 15.61)	13.15 (1986.3)	13.12 (11.58)	3.71 (2.05)
Interstitial lung disease	Respiratory, thoracic and mediastinal disorders	161	29.13 (24.91 - 34.06)	28.52 (4259.07)	28.39 (24.91)	4.83 (3.16)
Neuropathy peripheral	Nervous system disorders	131	11.47 (9.65 - 13.64)	11.29 (1228.07)	11.27 (9.75)	3.49 (1.83)
Anaemia	Blood and lymphatic system disorders	119	5.13 (4.28 - 6.14)	5.06 (388.54)	5.06 (4.35)	2.34 (0.67)
Decreased appetite	Metabolism and nutrition disorders	96	3.34 (2.73 - 4.08)	3.31 (155.02)	3.31 (2.79)	1.72 (0.06)
Stomatitis	Gastrointestinal disorders	92	12.24 (9.97 - 15.04)	12.1 (936.38)	12.08 (10.17)	3.59 (1.93)
Sepsis	Infections and infestations	89	6.7 (5.44 - 8.26)	6.63 (426.16)	6.63 (5.56)	2.73 (1.06)
Thrombocytopenia	Blood and lymphatic system disorders	78	6.05 (4.84 - 7.56)	6 (325.03)	5.99 (4.97)	2.58 (0.92)
Pleural effusion	Respiratory, thoracic and mediastinal disorders	57	8.02 (6.18 - 10.41)	7.97 (347.06)	7.96 (6.4)	2.99 (1.33)
Pulmonary embolism	Respiratory, thoracic and mediastinal disorders	49	4.35 (3.28 - 5.75)	4.32 (125.27)	4.32 (3.42)	2.11 (0.45)
Mucosal inflammation	General disorders and administration site conditions	47	15.51 (11.64 - 20.67)	15.42 (632.59)	15.39 (12.1)	3.94 (2.28)
Platelet count decreased	Investigations	42	3.24 (2.39 - 4.39)	3.23 (64.7)	3.23 (2.5)	1.69 (0.02)
Cardiac failure	Cardiac disorders	41	4.26 (3.13 - 5.79)	4.24 (101.55)	4.24 (3.28)	2.08 (0.42)
Hepatic function abnormal	Hepatobiliary disorders	39	9.5 (6.93 - 13.01)	9.45 (294.47)	9.44 (7.25)	3.24 (1.57)
Pancytopenia	Blood and lymphatic system disorders	35	5.51 (3.95 - 7.68)	5.49 (128.42)	5.48 (4.15)	2.45 (0.79)
Lymphopenia	Blood and lymphatic system disorders	31	18.14 (12.74 - 25.83)	18.07 (498.51)	18.02 (13.41)	4.17 (2.51)
Aspartate aminotransferase increased	Investigations	31	5.94 (4.17 - 8.45)	5.92 (126.64)	5.91 (4.4)	2.56 (0.9)
Pneumonitis	Respiratory, thoracic and mediastinal disorders	30	9.45 (6.6 - 13.53)	9.41 (225.38)	9.4 (6.96)	3.23 (1.57)
Alanine aminotransferase increased	Investigations	28	4.47 (3.09 - 6.48)	4.46 (75.16)	4.46 (3.27)	2.16 (0.49)
Respiratory failure	Respiratory, thoracic and mediastinal disorders	28	3.33 (2.3 - 4.83)	3.32 (45.42)	3.32 (2.43)	1.73 (0.06)
Septic shock	Infections and infestations	25	4.98 (3.36 - 7.37)	4.96 (79.14)	4.96 (3.57)	2.31 (0.64)
Hepatic failure	Hepatobiliary disorders	22	6.7 (4.41 - 10.19)	6.69 (106.3)	6.68 (4.71)	2.74 (1.07)
Hypokalaemia	Metabolism and nutrition disorders	21	3.94 (2.56 - 6.04)	3.93 (45.85)	3.93 (2.74)	1.97 (0.31)

Notably, the respiratory system manifested pleural effusion (n = 57), pulmonary embolism (n = 49) and respiratory failure (n = 28). Within the infections and infestations system, septic shock (n = 25) was reported. Additionally, the hepatic system exhibited hepatic failure (n = 22), and cardiac disorders manifested as cardiac failure (n = 41). Notably, none of these adverse events appear to have been previously reported in the product insert.

In addition, in Table 4, we show the top 30 PTs and their corresponding SOCs in descending order of ROR among the 95 positive adverse events. In addition to common hematologic toxicities, the top 3 AEs with the highest RORs were pseudocirrhosis (n = 8, ROR = 204.85), lymphangiosis carcinomatosa (n = 9, ROR = 73.31), peripheral motor neuropathy (n = 8, ROR = 50.53), which need to be taken seriously. It is worth noting that there are also some rare adverse events not reported in the drug label. First, in the category of injuries, poisonings, and procedural complications, recall phenomenon (n = 4, ROR = 42.96) has been observed. Second, in metabolic and nutritional disorders, fulminant type 1 diabetes mellitus (n = 5, ROR = 40.44) has been observed. In the nervous system, adverse events such as demyelinating polyneuropathy (n = 4, ROR = 34.41), vocal cord paralysis (n = 3, ROR = 14.51), and diplegia (n = 3, ROR = 12.99) have been reported. In the renal and urinary disorders, cystitis hemorrhagic (n = 8, ROR = 16.79) has been observed. In the vascular disorders, jugular vein thrombosis (n = 3, ROR = 14.47) has been noted. Although the number of these adverse events is small, they still deserve clinical attention.

**Table 4:** Top 30 PTs and their corresponding SOCs in descending order of ROR for positive adverse events.

Preferred Term (PT)	System Organ Class (SOC)	Number of reports	ROR (95% CI)	PRR (χ2)	EBGM (EBGM05)	IC (IC025)
Pseudocirrhosis	Hepatobiliary disorders	8	204.85 (101.25 - 414.43)	204.63 (1569.4)	198.14 (109.88)	7.63 (5.95)
Myelosuppression	Blood and lymphatic system disorders	291	109.68 (97.45 - 123.43)	105.43 (29611.3)	103.69 (93.93)	6.7 (5.03)
Lymphangiosis carcinomatosa	Neoplasms benign, malignant and unspecified (Incl cysts and polyps)	9	73.31 (37.98 - 141.49)	73.22 (633.65)	72.38 (41.75)	6.18 (4.51)
Febrile neutropenia	Blood and lymphatic system disorders	413	55.73 (50.44 - 61.56)	52.69 (20790.36)	52.26 (48.08)	5.71 (4.04)
Peripheral motor neuropathy	Nervous system disorders	8	50.53 (25.19 - 101.37)	50.48 (384.9)	50.08 (27.97)	5.65 (3.98)
Febrile infection	Infections and infestations	6	43.5 (19.48 - 97.13)	43.47 (247.21)	43.17 (22.04)	5.43 (3.76)
Recall phenomenon	Injury, poisoning and procedural complications	4	42.96 (16.07 - 114.89)	42.94 (162.73)	42.65 (18.73)	5.41 (3.74)
Fulminant type 1 diabetes mellitus	Metabolism and nutrition disorders	5	40.44 (16.78 - 97.46)	40.41 (190.95)	40.16 (19.24)	5.33 (3.66)
Neutrophil count decreased	Investigations	188	39.36 (34.04 - 45.51)	38.39 (6808.77)	38.16 (33.79)	5.25 (3.59)
Malignant pleural effusion	Respiratory, thoracic and mediastinal disorders	8	39.32 (19.61 - 78.82)	39.28 (296.55)	39.04 (21.81)	5.29 (3.62)
Neutropenia	Blood and lymphatic system disorders	556	36.04 (33.05 - 39.3)	33.42 (17434.22)	33.25 (30.93)	5.06 (3.39)
Leukopenia	Blood and lymphatic system disorders	195	34.47 (29.89 - 39.75)	33.59 (6137.75)	33.42 (29.66)	5.06 (3.4)
Demyelinating polyneuropathy	Nervous system disorders	4	34.41 (12.87 - 91.94)	34.39 (128.96)	34.2 (15.03)	5.1 (3.43)
Metastases to meninges	Neoplasms benign, malignant and unspecified (Incl cysts and polyps)	8	30.3 (15.12 - 60.71)	30.26 (225.29)	30.12 (16.84)	4.91 (3.25)
Interstitial lung disease	Respiratory, thoracic and mediastinal disorders	161	29.13 (24.91 - 34.06)	28.52 (4259.07)	28.39 (24.91)	4.83 (3.16)
Tumour pain	Neoplasms benign, malignant and unspecified (Incl cysts and polyps)	5	27.97 (11.61 - 67.34)	27.95 (129.34)	27.83 (13.34)	4.8 (3.13)
Malignant neoplasm progression	Neoplasms benign, malignant and unspecified (Incl cysts and polyps)	299	25.02 (22.28 - 28.09)	24.05 (6592.04)	23.96 (21.75)	4.58 (2.92)
Pharyngeal ulceration	Respiratory, thoracic and mediastinal disorders	4	24.92 (9.33 - 66.56)	24.91 (91.44)	24.82 (10.91)	4.63 (2.96)
Tumour necrosis	Neoplasms benign, malignant and unspecified (Incl cysts and polyps)	3	24.29 (7.81 - 75.49)	24.28 (66.7)	24.19 (9.37)	4.6 (2.93)
Neutropenic sepsis	Infections and infestations	17	19.52 (12.12 - 31.43)	19.47 (297.03)	19.42 (13.03)	4.28 (2.61)
Hyperpyrexia	General disorders and administration site conditions	9	19.42 (10.09 - 37.37)	19.4 (156.56)	19.34 (11.18)	4.27 (2.61)
Lymphopenia	Blood and lymphatic system disorders	31	18.14 (12.74 - 25.83)	18.07 (498.51)	18.02 (13.41)	4.17 (2.51)
Cystitis haemorrhagic	Renal and urinary disorders	8	16.79 (8.38 - 33.61)	16.77 (118.33)	16.73 (9.36)	4.06 (2.4)
Peripheral sensory neuropathy	Nervous system disorders	11	15.98 (8.84 - 28.89)	15.96 (153.82)	15.92 (9.7)	3.99 (2.33)
Mucosal inflammation	General disorders and administration site conditions	47	15.51 (11.64 - 20.67)	15.42 (632.59)	15.39 (12.1)	3.94 (2.28)
Hypertransaminasaemia	Hepatobiliary disorders	11	14.54 (8.04 - 26.29)	14.52 (138.19)	14.49 (8.83)	3.86 (2.19)
Vocal cord paralysis	Nervous system disorders	3	14.51 (4.67 - 45.05)	14.5 (37.62)	14.47 (5.61)	3.85 (2.19)
Jugular vein thrombosis	Vascular disorders	3	14.47 (4.66 - 44.94)	14.47 (37.52)	14.43 (5.59)	3.85 (2.18)
White blood cell count decreased	Investigations	177	13.44 (11.58 - 15.61)	13.15 (1986.3)	13.12 (11.58)	3.71 (2.05)
Diplegia	Nervous system disorders	3	12.99 (4.18 - 40.33)	12.99 (33.12)	12.96 (5.02)	3.7 (2.03)

It is important to note that the 95 positive PTs that met the four algorithms (S3 Table) did not include adverse events such as prolonged QT interval, alopecia, nausea, and constipation, which were included in the drug insert.

### Time-to-onset analysis

**Fig 4:**
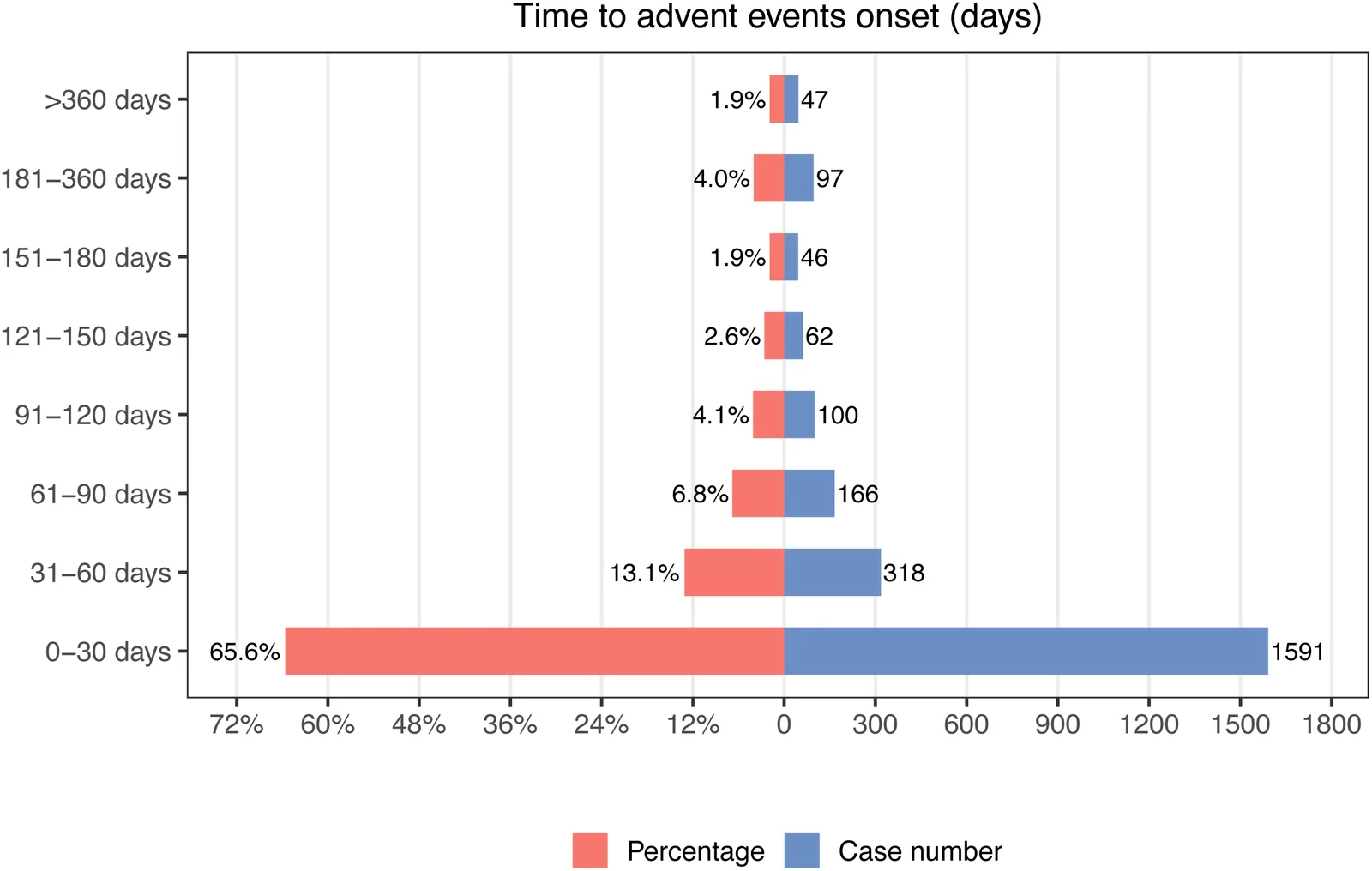
Distribution of time to onset of eribulin-associated adverse events.

A total of 2427 reports contained sufficient information in the THER file to calculate time to onset. The mean time to onset was 49.4 ± 108.3 days, and the median was 13 days (interquartile range [IQR]: 7–49 days). Fig 4 illustrates the number of reported cases and the percentage distribution of eribulin-associated AEs across various time intervals. Primarily, the majority of eribulin-related AEs emerged within 30 days post-dosing (n = 1591, 65.6%). Secondly, there was an increase in the number of AEs in the 181-360s day timeframe compared to the preceding period (i.e., within 151–180 days), rising from 1.9% to 4%. Notably, despite this escalation, the overall proportion of reports of AEs demonstrated a gradual decline over time subsequent to the initial 30 days of eribulin administration.

**Fig 5:**
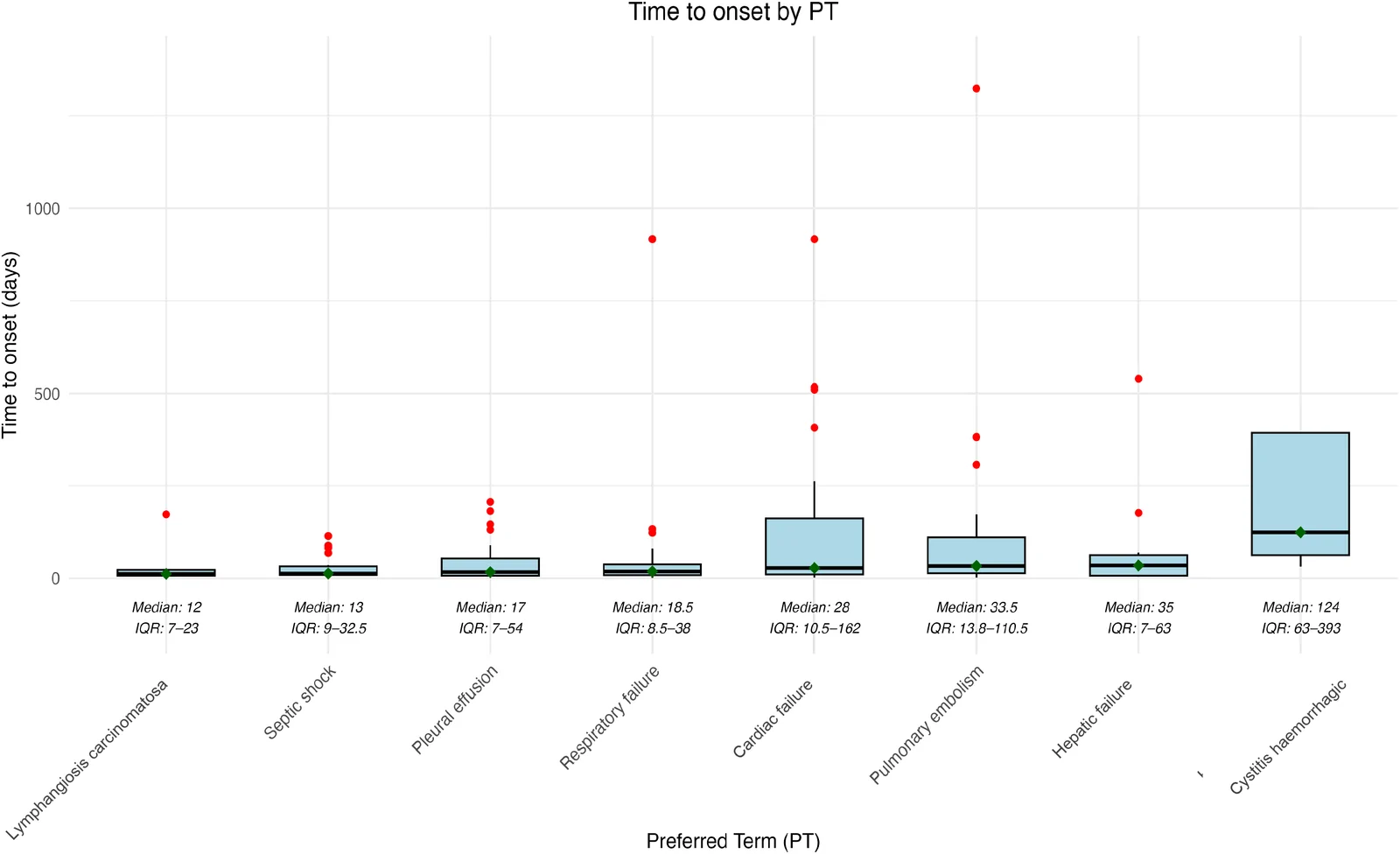
The box plot illustrates the distribution of time to onset for the adverse events newly identified in this study, showing only those with ≥5 reported cases.

Fig 5 shows the time to onset for newly identified adverse events with ≥5 cases. As can be seen, lymphangiosis carcinomatosa, septic shock, pleural effusion and respiratory failure occurred earlier (median < 28 days), whereas cardiac failure, pulmonary embolism, hepatic failure and cystitis haemorrhagic occurred later (median ≥ 28 days). Furthermore, cystitis haemorrhagic had the widest interquartile range (IQR: 63–393 days), indicating greater variability. The aforementioned comparisons are descriptive in nature and do not establish any differences among these preferred terms with respect to incidence or risk.

## Discussion

Eribulin, a fully synthetic analogue of halichondrin B, is a microtubule-targeting agent that uniquely inhibits microtubule dynamics. It exerts non-mitotic effects on tumors, influencing epithelial-mesenchymal transition and tumor vasculature. Its hematologic toxicity profile is well-documented, yet a comprehensive safety analysis remains lacking. In recent times, eribulin has shown synergy with tumor immunotherapy and remains a treatment option for mBC. Notably, a study utilizing eribulin as the payload in an antibody-drug conjugate (ADC) [19], termed MORAb-202, exhibited robust anti-tumor efficacy in a triple-negative breast cancer (TNBC) nude mouse model [20]. MORAb-202 has successfully completed a Phase 1 study in Japan [21], demonstrating antitumor activity against folate receptor α-positive tumors and exhibiting overall good tolerability. Concurrently, additional clinical research programs on MORAb-202 are underway globally.

These studies highlight eribulin’s versatile applications and underscore the importance for a thorough investigation of its adverse events. Using a real-world pharmacovigilance database, this study aims to systematically analyze eribulin-associated adverse events to mitigate future risks and improve clinical safety.

Our investigation revealed a heightened proportion of reports of adverse events among female patients administered eribulin, potentially attributable to eribulin’s predominant use in individuals with mBC. Notably, the number of adverse events was elevated in patients aged 35–59 years compared to those older than 60 years, aligning with the reported age of onset for metastatic BC in clinical studies, predominantly involving patients younger than 60 years [22–24]. Furthermore, the pharmacovigilance data obtained in this study were predominantly sourced from physicians’ uploads, thus assuring the reliability of our findings. The volume of reported cases in Japan underscores the nation’s emphasis on documenting eribulin-associated adverse reactions. It is worth highlighting that, despite a temporary decline, the number of eribulin-related adverse event reports has resurged since 2020, underscoring the need for increased vigilance.

Reports associated with eribulin are primarily linked to blood and lymphatic system disorders, as well as hepatobiliary disorders. Specifically, these disorders encompass neutropenia, febrile neutropenia, and myelosuppression, consistent with those in drug labeling and clinical study reports [25–27]. Eribulin’s antitumor mechanism hinges on its broad-spectrum antimitotic activity, which may impact normal blood cells. Notably, patients treated with eribulin have typically undergone multiple lines of chemotherapy, with residual effects that may exacerbate hematotoxicity [28]. Furthermore, hepatobiliary AEs include hepatic function abnormalities and hepatic failure, among others. Exposure to eribulin is increased in patients with hepatic impairment [29], necessitating a dosage reduction for patients with abnormal liver function [30]. However, the dosage form does not explicitly mention adverse reactions that may lead to hepatic failure. A case report detailing acute hepatic failure in a patient with breast cancer hepatic metastases treated with eribulin serves as a cautionary tale for clinicians [31].

Based on the analysis conducted at the PT level, the elevated number of AEs during malignant tumor progression may be attributed to patients’ suboptimal response to eribulin or the premature treatment termination. Furthermore, neurologic-related AEs, notably peripheral neuropathy, are significant in clinical studies involving eribulin. This adverse reaction involves sensory and motor disturbances, including numbness, tingling sensations, pain, and extremity weakness [32]. A clinical study conducted in Italy enrolled 170 patients for safety assessment, revealing that neurotoxicity occurred in 14.7% of these patients [33]. Another study confirmed that 7.6% of patients reduced, interrupted, or delayed eribulin due to peripheral neurotoxicity, and 6.7% discontinued it entirely [34].

Notably, our findings indicate that multiple respiratory-related AEs, such as pleural effusion, pulmonary embolism and respiratory failure, were omitted from the drug labeling, highlighting the need for enhanced lung imaging monitoring. Among respiratory-related AEs, a limited number of clinical trials have reported interstitial lung disease [21,35,36]. The underlying mechanism remains unclear, but it may be associated with eribulin’s pulmonary toxicity and impaired immune function.

Granulocytopenia can lead to fever and infection, including sepsis and septic shock, associated with reduced neutrophils and weakened immunity. Though rare, these outcomes can be fatal. For instance, a phase 1 study of balixafortide plus eribulin in heavily pretreated recurrent metastatic breast cancer reported one patient (1/56) dying from infectious shock [37]. Therefore, clinicians should monitor blood counts and neutrophil levels, considering dose reduction or discontinuation in severe cases. Furthermore, our study reported cardiac AEs such as cardiac failure, also not mentioned in the drug label.

Pseudocirrhosis is particularly noteworthy in AEs exhibiting high ROR values. A recent case report described pseudocirrhosis during eribulin treatment in five patients, leading to poor prognosis despite initial tumor shrinkage [38]. This could be associated with nodular regenerative hyperplasia, connective tissue proliferation/fibrosis, tumor infiltration, or drug-induced hepatotoxicity [39]. Our study revealed that eribulin may precipitate AEs in patients with fulminant type 1 diabetes mellitus. A Phase I clinical trial investigating eribulin in conjunction with everolimus for triple-negative mBC observed hyperglycemic AEs in 3 out of 27 patients [40]; however, it was unclear whether these were due to eribulin or drug interactions. Furthermore, several AEs, including vocal cord paralysis, diplegia, cystitis haemorrhagic, and jugular vein thrombosis, have not been documented in clinical studies. These unreported AEs necessitate further study to elucidate their mechanisms.

The median time to onset of eribulin-related AEs was 13 days, with the majority occurring within 30 days of treatment, defining an early high-risk period. However, the large standard deviation (108.3 days) and a late rise in number at 180–360 days may indicate interindividual variability and the potential for delayed toxicity. We also compiled data on the onset times of certain AEs not mentioned in the product information, and found that these findings underscore the necessity of close monitoring during the first month, continued follow-up beyond six months, and vigilance regarding highly variable adverse events such as cystitis haemorrhagic.

However, there are some limitations of this study. First, FAERS is a spontaneous-reporting database and is subject to underreporting, duplicate reporting, reporting bias, and missing or inaccurate information. Second, about 47% of eribulin-related AEs were from Japan and China, which may limit generalizability to other regions. Third, most FAERS-based studies are retrospective, introducing bias; disproportionality signal detection indicates reporting association, not causality. Finally, dose–response analysis was not feasible because dose and treatment-duration data were frequently missing or uninterpretable, units were inconsistent, and report counts for individual events were small.

Future research should integrate FAERS data with other real-world data to obtain more precise clinical information. For example, in clinical practice, combining evidence from randomised controlled trials and real-world cohort studies can help to establish a causal relationship between eribulin and adverse events, and to assess dose-response relationships more reliably.

## Conclusion

In this study, the analysis identified eribulin-associated disproportionality signals, with the strongest and most consistent signals involving hematologic and lymphatic system disorders. In addition, respiratory failure, septic shock, pseudo-cirrhosis, and type 1 diabetes mellitus were not mentioned in the drug labeling, which need to be further strengthened in clinical monitoring and reporting. In the future, actively conducting clinical studies with large samples for more comprehensive safety assessment will be the key research direction.

## Supporting information

S1 Table 2 × 2 Columnar Table. (DOCX)

S2 Table Four algorithms for disproportionality analysis and their associated positivity thresholds. (DOCX)

S3 Table 95 positive disproportionality signals and associated SOCs. (DOCX)

S4 Data Raw data for Figs and Tables in this manuscript. (XLSX)
