# Supplementary material for: Safety of eribulin: A real-world study based on a pharmacovigilance database
Source: PLoS One. 2026 Sep 17;21(9):e0356800. doi: 10.1371/journal.pone.0356800 (PMC13585259; doi:10.1371/journal.pone.0356800)
Supplement: Supplementary file 1 [file pone.0356800.s001.docx]

**S1 Table.** 2×2 Columnar Table.

|  | Target drug’s ADEs | Other drugs’ ADEs | Total |
| --- | --- | --- | --- |
| Target drug | a | b | a + b |
| Other drugs | c | d | c + d |
| Total | a + c | b + d | N = a + b + c + d |

Notes: a, number of cases with target adverse events for target drugs; b, number of cases with other adverse events for target drugs; c, number of cases with the target adverse event for other drugs; d, number of cases with other adverse event for other drugs.
