# Supplementary material for: Safety of eribulin: A real-world study based on a pharmacovigilance database
Source: PLoS One. 2026 Sep 17;21(9):e0356800. doi: 10.1371/journal.pone.0356800 (PMC13585259; doi:10.1371/journal.pone.0356800)
Supplement: Supplementary file 2 [file pone.0356800.s002.docx]

**S2 Table.** Four algorithms for disproportionality analysis and their associated positivity thresholds.

| **Algorithms** | **Target drug’s ADEs** | **Positive conditions** |
| --- | --- | --- |
| **ROR** | $ROR=\frac{\mathrm{ad}}{\mathrm{bc}}$ | a ≥ 3  95%CI (lower limit) > 1 |
|  | $SE(lnROR)=\sqrt{(\frac{1}{a}+\frac{1}{b}+\frac{1}{c}+\frac{1}{d}})$ |  |
|  | $95\%CI=e^{ln(ROR)\pm1.96\sqrt{(\frac{1}{a}+\frac{1}{b}+\frac{1}{c}+\frac{1}{d}})}$ |  |
| **PRR** | $PRR=\frac{a / (a+b)}{c / (c+d)}$ | a ≥ 3  PRR ≥ 2  $\chi^{2}$≥4 |
|  | $\chi^{2} =\frac{{(ad-bc)}^{2} (a+b+c+d)}{(a+b)(c+d)(a+c)(b+d)}$ |  |
| **BCPNN** | $IC= {log}_{2}\frac{a(a+b+c+d)}{(a+b)(a+c)}$ | IC025>0 |
|  | $95\%CI=E\left( \mathrm{IC} \right)\pm2 \sqrt{V(IC)}$ |  |
| **MGPS** | $EBGM=\frac{a(a+b+c+d)}{\left( a+c \right)(a+b)}$ | EBGM05>2 |
|  | $SE(lnEBGM)=\sqrt{(\frac{1}{a}+\frac{1}{b}+\frac{1}{c}+\frac{1}{d}})$ |  |
|  | $95\%CI= e^{\ln\left( EBGM \right)\pm1.96\sqrt{(\frac{1}{a}+\frac{1}{b}+\frac{1}{c}+\frac{1}{d}})}$ |  |

Notes: 95%CI, 95% confidence interval; $\chi^{2}$, chi-squared; IC, information component; IC025, the lower limit of 95% CI; E(IC), the expectation of IC; V(IC), the variance of IC; EBGM, Empirical Bayesian Geometric Mean; EBGM05, the lower limit of 95% CI of EBGM.
