# Supplementary material for: Safety of eribulin: A real-world study based on a pharmacovigilance database
Source: PLoS One. 2026 Sep 17;21(9):e0356800. doi: 10.1371/journal.pone.0356800 (PMC13585259; doi:10.1371/journal.pone.0356800)
Supplement: Supplementary file 3 [file pone.0356800.s003.docx]

**S3 Table.** 95 positive AEs and associated SOCs.

| Preferred Term (PT) | System Organ Class (SOC) | Number of reports | ROR (95% Cl) | PRR (χ2) | EBGM (EBGM05) | IC (IC025) |
| --- | --- | --- | --- | --- | --- | --- |
| Neutropenia | Blood and lymphatic system disorders | 556 | 36.04 ( 33.05 - 39.3 ) | 33.42 ( 17434.22 ) | 33.25 ( 30.93 ) | 5.06 ( 3.39 ) |
| Febrile neutropenia | Blood and lymphatic system disorders | 413 | 55.73 ( 50.44 - 61.56 ) | 52.69 ( 20790.36 ) | 52.26 ( 48.08 ) | 5.71 ( 4.04 ) |
| Malignant neoplasm progression | Neoplasms benign, malignant and unspecified (incl cysts and polyps) | 299 | 25.02 ( 22.28 - 28.09 ) | 24.05 ( 6592.04 ) | 23.96 ( 21.75 ) | 4.58 ( 2.92 ) |
| Myelosuppression | Blood and lymphatic system disorders | 291 | 109.68 ( 97.45 - 123.43 ) | 105.43 ( 29611.3 ) | 103.69 ( 93.93 ) | 6.7 ( 5.03 ) |
| Leukopenia | Blood and lymphatic system disorders | 195 | 34.47 ( 29.89 - 39.75 ) | 33.59 ( 6137.75 ) | 33.42 ( 29.66 ) | 5.06 ( 3.4 ) |
| Neutrophil count decreased | Investigations | 188 | 39.36 ( 34.04 - 45.51 ) | 38.39 ( 6808.77 ) | 38.16 ( 33.79 ) | 5.25 ( 3.59 ) |
| Pyrexia | General disorders and administration site conditions | 183 | 4.48 ( 3.87 - 5.19 ) | 4.39 ( 481.81 ) | 4.39 ( 3.88 ) | 2.13 ( 0.47 ) |
| White blood cell count decreased | Investigations | 177 | 13.44 ( 11.58 - 15.61 ) | 13.15 ( 1986.3 ) | 13.12 ( 11.58 ) | 3.71 ( 2.05 ) |
| Interstitial lung disease | Respiratory, thoracic and mediastinal disorders | 161 | 29.13 ( 24.91 - 34.06 ) | 28.52 ( 4259.07 ) | 28.39 ( 24.91 ) | 4.83 ( 3.16 ) |
| Neuropathy peripheral | Nervous system disorders | 131 | 11.47 ( 9.65 - 13.64 ) | 11.29 ( 1228.07 ) | 11.27 ( 9.75 ) | 3.49 ( 1.83 ) |
| Anaemia | Blood and lymphatic system disorders | 119 | 5.13 ( 4.28 - 6.14 ) | 5.06 ( 388.54 ) | 5.06 ( 4.35 ) | 2.34 ( 0.67 ) |
| Decreased appetite | Metabolism and nutrition disorders | 96 | 3.34 ( 2.73 - 4.08 ) | 3.31 ( 155.02 ) | 3.31 ( 2.79 ) | 1.72 ( 0.06 ) |
| Stomatitis | Gastrointestinal disorders | 92 | 12.24 ( 9.97 - 15.04 ) | 12.1 ( 936.38 ) | 12.08 ( 10.17 ) | 3.59 ( 1.93 ) |
| Sepsis | Infections and infestations | 89 | 6.7 ( 5.44 - 8.26 ) | 6.63 ( 426.16 ) | 6.63 ( 5.56 ) | 2.73 ( 1.06 ) |
| Thrombocytopenia | Blood and lymphatic system disorders | 78 | 6.05 ( 4.84 - 7.56 ) | 6 ( 325.03 ) | 5.99 ( 4.97 ) | 2.58 ( 0.92 ) |
| Pleural effusion | Respiratory, thoracic and mediastinal disorders | 57 | 8.02 ( 6.18 - 10.41 ) | 7.97 ( 347.06 ) | 7.96 ( 6.4 ) | 2.99 ( 1.33 ) |
| Pulmonary embolism | Respiratory, thoracic and mediastinal disorders | 49 | 4.35 ( 3.28 - 5.75 ) | 4.32 ( 125.27 ) | 4.32 ( 3.42 ) | 2.11 ( 0.45 ) |
| Mucosal inflammation | General disorders and administration site conditions | 47 | 15.51 ( 11.64 - 20.67 ) | 15.42 ( 632.59 ) | 15.39 ( 12.1 ) | 3.94 ( 2.28 ) |
| Platelet count decreased | Investigations | 42 | 3.24 ( 2.39 - 4.39 ) | 3.23 ( 64.7 ) | 3.23 ( 2.5 ) | 1.69 ( 0.02 ) |
| Cardiac failure | Cardiac disorders | 41 | 4.26 ( 3.13 - 5.79 ) | 4.24 ( 101.55 ) | 4.24 ( 3.28 ) | 2.08 ( 0.42 ) |
| Hepatic function abnormal | Hepatobiliary disorders | 39 | 9.5 ( 6.93 - 13.01 ) | 9.45 ( 294.47 ) | 9.44 ( 7.25 ) | 3.24 ( 1.57 ) |
| Pancytopenia | Blood and lymphatic system disorders | 35 | 5.51 ( 3.95 - 7.68 ) | 5.49 ( 128.42 ) | 5.48 ( 4.15 ) | 2.45 ( 0.79 ) |
| Lymphopenia | Blood and lymphatic system disorders | 31 | 18.14 ( 12.74 - 25.83 ) | 18.07 ( 498.51 ) | 18.02 ( 13.41 ) | 4.17 ( 2.51 ) |
| Aspartate aminotransferase increased | Investigations | 31 | 5.94 ( 4.17 - 8.45 ) | 5.92 ( 126.64 ) | 5.91 ( 4.4 ) | 2.56 ( 0.9 ) |
| Pneumonitis | Respiratory, thoracic and mediastinal disorders | 30 | 9.45 ( 6.6 - 13.53 ) | 9.41 ( 225.38 ) | 9.4 ( 6.96 ) | 3.23 ( 1.57 ) |
| Respiratory failure | Respiratory, thoracic and mediastinal disorders | 28 | 3.33 ( 2.3 - 4.83 ) | 3.32 ( 45.42 ) | 3.32 ( 2.43 ) | 1.73 ( 0.06 ) |
| Alanine aminotransferase increased | Investigations | 28 | 4.47 ( 3.09 - 6.48 ) | 4.46 ( 75.16 ) | 4.46 ( 3.27 ) | 2.16 ( 0.49 ) |
| Septic shock | Infections and infestations | 25 | 4.98 ( 3.36 - 7.37 ) | 4.96 ( 79.14 ) | 4.96 ( 3.57 ) | 2.31 ( 0.64 ) |
| Hepatic failure | Hepatobiliary disorders | 22 | 6.7 ( 4.41 - 10.19 ) | 6.69 ( 106.3 ) | 6.68 ( 4.71 ) | 2.74 ( 1.07 ) |
| Hypokalaemia | Metabolism and nutrition disorders | 21 | 3.94 ( 2.56 - 6.04 ) | 3.93 ( 45.85 ) | 3.93 ( 2.74 ) | 1.97 ( 0.31 ) |
| Pneumothorax | Respiratory, thoracic and mediastinal disorders | 21 | 10.76 ( 7.01 - 16.52 ) | 10.74 ( 185.12 ) | 10.72 ( 7.49 ) | 3.42 ( 1.76 ) |
| Hyperglycaemia | Metabolism and nutrition disorders | 20 | 4.87 ( 3.14 - 7.55 ) | 4.86 ( 61.21 ) | 4.85 ( 3.36 ) | 2.28 ( 0.61 ) |
| Neutropenic sepsis | Infections and infestations | 17 | 19.52 ( 12.12 - 31.43 ) | 19.47 ( 297.03 ) | 19.42 ( 13.03 ) | 4.28 ( 2.61 ) |
| Polyneuropathy | Nervous system disorders | 16 | 11.32 ( 6.93 - 18.5 ) | 11.3 ( 149.97 ) | 11.28 ( 7.48 ) | 3.5 ( 1.83 ) |
| Ascites | Gastrointestinal disorders | 15 | 4.33 ( 2.61 - 7.19 ) | 4.32 ( 38.32 ) | 4.32 ( 2.83 ) | 2.11 ( 0.45 ) |
| Mouth ulceration | Gastrointestinal disorders | 14 | 5.73 ( 3.39 - 9.68 ) | 5.72 ( 54.47 ) | 5.71 ( 3.68 ) | 2.51 ( 0.85 ) |
| Disseminated intravascular coagulation | Blood and lymphatic system disorders | 13 | 8.85 ( 5.14 - 15.26 ) | 8.84 ( 90.25 ) | 8.83 ( 5.6 ) | 3.14 ( 1.48 ) |
| Hepatotoxicity | Hepatobiliary disorders | 13 | 5.01 ( 2.91 - 8.64 ) | 5.01 ( 41.65 ) | 5 ( 3.17 ) | 2.32 ( 0.66 ) |
| Drug-induced liver injury | Hepatobiliary disorders | 13 | 3.49 ( 2.02 - 6.01 ) | 3.48 ( 23.02 ) | 3.48 ( 2.21 ) | 1.8 ( 0.13 ) |
| Metastases to central nervous system | Neoplasms benign, malignant and unspecified (incl cysts and polyps) | 12 | 7.91 ( 4.49 - 13.95 ) | 7.9 ( 72.27 ) | 7.89 ( 4.91 ) | 2.98 ( 1.31 ) |
| Palmar-plantar erythrodysesthesia syndrome | Skin and subcutaneous tissue disorders | 11 | 3.7 ( 2.05 - 6.69 ) | 3.7 ( 21.63 ) | 3.69 ( 2.25 ) | 1.89 ( 0.22 ) |
| Hypertransaminasaemia | Hepatobiliary disorders | 11 | 14.54 ( 8.04 - 26.29 ) | 14.52 ( 138.19 ) | 14.49 ( 8.83 ) | 3.86 ( 2.19 ) |
| Peripheral sensory neuropathy | Nervous system disorders | 11 | 15.98 ( 8.84 - 28.89 ) | 15.96 ( 153.82 ) | 15.92 ( 9.7 ) | 3.99 ( 2.33 ) |
| Blood bilirubin increased | Investigations | 11 | 3.86 ( 2.13 - 6.97 ) | 3.85 ( 23.23 ) | 3.85 ( 2.35 ) | 1.95 ( 0.28 ) |
| Hepatic cirrhosis | Hepatobiliary disorders | 10 | 4.63 ( 2.49 - 8.61 ) | 4.62 ( 28.4 ) | 4.62 ( 2.75 ) | 2.21 ( 0.54 ) |
| Bacteraemia | Infections and infestations | 10 | 7.26 ( 3.9 - 13.51 ) | 7.25 ( 53.87 ) | 7.25 ( 4.31 ) | 2.86 ( 1.19 ) |
| Stevens-johnson syndrome | Skin and subcutaneous tissue disorders | 9 | 4.15 ( 2.16 - 7.98 ) | 4.14 ( 21.46 ) | 4.14 ( 2.4 ) | 2.05 ( 0.38 ) |
| Lymphangiosis carcinomatosa | Neoplasms benign, malignant and unspecified (incl cysts and polyps) | 9 | 73.31 ( 37.98 - 141.49 ) | 73.22 ( 633.65 ) | 72.38 ( 41.75 ) | 6.18 ( 4.51 ) |
| Hyperpyrexia | General disorders and administration site conditions | 9 | 19.42 ( 10.09 - 37.37 ) | 19.4 ( 156.56 ) | 19.34 ( 11.18 ) | 4.27 ( 2.61 ) |
| Agranulocytosis | Blood and lymphatic system disorders | 9 | 4.34 ( 2.26 - 8.35 ) | 4.34 ( 23.1 ) | 4.34 ( 2.51 ) | 2.12 ( 0.45 ) |
| Ejection fraction decreased | Investigations | 8 | 4.4 ( 2.2 - 8.81 ) | 4.4 ( 21.01 ) | 4.4 ( 2.46 ) | 2.14 ( 0.47 ) |
| Cystitis haemorrhagic | Renal and urinary disorders | 8 | 16.79 ( 8.38 - 33.61 ) | 16.77 ( 118.33 ) | 16.73 ( 9.36 ) | 4.06 ( 2.4 ) |
| Oral candidiasis | Infections and infestations | 8 | 5.38 ( 2.69 - 10.76 ) | 5.37 ( 28.46 ) | 5.37 ( 3.01 ) | 2.42 ( 0.76 ) |
| Metastases to meninges | Neoplasms benign, malignant and unspecified (incl cysts and polyps) | 8 | 30.3 ( 15.12 - 60.71 ) | 30.26 ( 225.29 ) | 30.12 ( 16.84 ) | 4.91 ( 3.25 ) |
| Pneumocystis jirovecii pneumonia | Infections and infestations | 8 | 6.16 ( 3.08 - 12.32 ) | 6.15 ( 34.47 ) | 6.14 ( 3.44 ) | 2.62 ( 0.95 ) |
| Malignant pleural effusion | Respiratory, thoracic and mediastinal disorders | 8 | 39.32 ( 19.61 - 78.82 ) | 39.28 ( 296.55 ) | 39.04 ( 21.81 ) | 5.29 ( 3.62 ) |
| Metastases to liver | Neoplasms benign, malignant and unspecified (incl cysts and polyps) | 8 | 3.75 ( 1.88 - 7.51 ) | 3.75 ( 16.13 ) | 3.75 ( 2.1 ) | 1.91 ( 0.24 ) |
| Peripheral motor neuropathy | Nervous system disorders | 8 | 50.53 ( 25.19 - 101.37 ) | 50.48 ( 384.9 ) | 50.08 ( 27.97 ) | 5.65 ( 3.98 ) |
| Neurotoxicity | Nervous system disorders | 8 | 3.87 ( 1.93 - 7.74 ) | 3.87 ( 16.99 ) | 3.86 ( 2.16 ) | 1.95 ( 0.28 ) |
| Generalised oedema | General disorders and administration site conditions | 8 | 5.94 ( 2.97 - 11.89 ) | 5.94 ( 32.83 ) | 5.93 ( 3.32 ) | 2.57 ( 0.9 ) |
| Hypercalcaemia | Metabolism and nutrition disorders | 8 | 5.44 ( 2.72 - 10.89 ) | 5.44 ( 28.96 ) | 5.43 ( 3.04 ) | 2.44 ( 0.78 ) |
| Pseudocirrhosis | Hepatobiliary disorders | 8 | 204.85 ( 101.25 - 414.43 ) | 204.63 ( 1569.4 ) | 198.14 ( 109.88 ) | 7.63 ( 5.95 ) |
| Tumour lysis syndrome | Metabolism and nutrition disorders | 7 | 6.77 ( 3.23 - 14.22 ) | 6.77 ( 34.38 ) | 6.76 ( 3.64 ) | 2.76 ( 1.09 ) |
| Hypophosphataemia | Metabolism and nutrition disorders | 6 | 6.73 ( 3.02 - 14.98 ) | 6.72 ( 29.2 ) | 6.72 ( 3.44 ) | 2.75 ( 1.08 ) |
| Haematotoxicity | Blood and lymphatic system disorders | 6 | 5.51 ( 2.47 - 12.27 ) | 5.51 ( 22.11 ) | 5.5 ( 2.81 ) | 2.46 ( 0.79 ) |
| Hyperbilirubinaemia | Hepatobiliary disorders | 6 | 5.01 ( 2.25 - 11.16 ) | 5.01 ( 19.24 ) | 5.01 ( 2.56 ) | 2.32 ( 0.66 ) |
| Enterocolitis | Gastrointestinal disorders | 6 | 9.04 ( 4.06 - 20.13 ) | 9.03 ( 42.79 ) | 9.02 ( 4.61 ) | 3.17 ( 1.51 ) |
| Extravasation | General disorders and administration site conditions | 6 | 12.94 ( 5.8 - 28.83 ) | 12.93 ( 65.89 ) | 12.9 ( 6.6 ) | 3.69 ( 2.02 ) |
| Febrile infection | Infections and infestations | 6 | 43.5 ( 19.48 - 97.13 ) | 43.47 ( 247.21 ) | 43.17 ( 22.04 ) | 5.43 ( 3.76 ) |
| Granulocytopenia | Blood and lymphatic system disorders | 5 | 7.83 ( 3.26 - 18.83 ) | 7.83 ( 29.74 ) | 7.82 ( 3.75 ) | 2.97 ( 1.3 ) |
| Tumour pain | Neoplasms benign, malignant and unspecified (incl cysts and polyps) | 5 | 27.97 ( 11.61 - 67.34 ) | 27.95 ( 129.34 ) | 27.83 ( 13.34 ) | 4.8 ( 3.13 ) |
| Hepatic encephalopathy | Nervous system disorders | 5 | 4.43 ( 1.84 - 10.65 ) | 4.43 ( 13.26 ) | 4.43 ( 2.12 ) | 2.15 ( 0.48 ) |
| Hydronephrosis | Renal and urinary disorders | 5 | 5.76 ( 2.4 - 13.85 ) | 5.76 ( 19.65 ) | 5.76 ( 2.76 ) | 2.52 ( 0.86 ) |
| Urosepsis | Infections and infestations | 5 | 4.45 ( 1.85 - 10.7 ) | 4.45 ( 13.37 ) | 4.45 ( 2.14 ) | 2.15 ( 0.49 ) |
| Embolism | Vascular disorders | 5 | 5.24 ( 2.18 - 12.6 ) | 5.24 ( 17.13 ) | 5.23 ( 2.51 ) | 2.39 ( 0.72 ) |
| Fulminant type 1 diabetes mellitus | Metabolism and nutrition disorders | 5 | 40.44 ( 16.78 - 97.46 ) | 40.41 ( 190.95 ) | 40.16 ( 19.24 ) | 5.33 ( 3.66 ) |
| Febrile bone marrow aplasia | Blood and lymphatic system disorders | 4 | 7.96 ( 2.98 - 21.22 ) | 7.95 ( 24.29 ) | 7.94 ( 3.5 ) | 2.99 ( 1.32 ) |
| Meningitis | Infections and infestations | 4 | 5.23 ( 1.96 - 13.94 ) | 5.23 ( 13.66 ) | 5.22 ( 2.3 ) | 2.38 ( 0.72 ) |
| Demyelinating polyneuropathy | Nervous system disorders | 4 | 34.41 ( 12.87 - 91.94 ) | 34.39 ( 128.96 ) | 34.2 ( 15.03 ) | 5.1 ( 3.43 ) |
| Pharyngeal ulceration | Respiratory, thoracic and mediastinal disorders | 4 | 24.92 ( 9.33 - 66.56 ) | 24.91 ( 91.44 ) | 24.82 ( 10.91 ) | 4.63 ( 2.96 ) |
| Metabolic encephalopathy | Nervous system disorders | 4 | 11.58 ( 4.34 - 30.89 ) | 11.57 ( 38.57 ) | 11.55 ( 5.08 ) | 3.53 ( 1.86 ) |
| Neutrophilia | Blood and lymphatic system disorders | 4 | 5.8 ( 2.18 - 15.47 ) | 5.8 ( 15.87 ) | 5.79 ( 2.55 ) | 2.53 ( 0.87 ) |
| Recall phenomenon | Injury, poisoning and procedural complications | 4 | 42.96 ( 16.07 - 114.89 ) | 42.94 ( 162.73 ) | 42.65 ( 18.73 ) | 5.41 ( 3.74 ) |
| Tumour haemorrhage | Neoplasms benign, malignant and unspecified (incl cysts and polyps) | 4 | 11.62 ( 4.36 - 31.01 ) | 11.62 ( 38.75 ) | 11.6 ( 5.1 ) | 3.54 ( 1.87 ) |
| Vocal cord paralysis | Nervous system disorders | 3 | 14.51 ( 4.67 - 45.05 ) | 14.5 ( 37.62 ) | 14.47 ( 5.61 ) | 3.85 ( 2.19 ) |
| Jaundice cholestatic | Hepatobiliary disorders | 3 | 7.89 ( 2.54 - 24.5 ) | 7.89 ( 18.03 ) | 7.88 ( 3.06 ) | 2.98 ( 1.31 ) |
| Diplegia | Nervous system disorders | 3 | 12.99 ( 4.18 - 40.33 ) | 12.99 ( 33.12 ) | 12.96 ( 5.02 ) | 3.7 ( 2.03 ) |
| Systemic inflammatory response syndrome | General disorders and administration site conditions | 3 | 6.32 ( 2.04 - 19.62 ) | 6.32 ( 13.42 ) | 6.32 ( 2.45 ) | 2.66 ( 0.99 ) |
| Nephritis | Renal and urinary disorders | 3 | 7.76 ( 2.5 - 24.08 ) | 7.76 ( 17.64 ) | 7.75 ( 3 ) | 2.95 ( 1.29 ) |
| Dysaesthesia | Nervous system disorders | 3 | 8.76 ( 2.82 - 27.19 ) | 8.76 ( 20.59 ) | 8.75 ( 3.39 ) | 3.13 ( 1.46 ) |
| Cardiac tamponade | Cardiac disorders | 3 | 5.36 ( 1.73 - 16.62 ) | 5.35 ( 10.61 ) | 5.35 ( 2.07 ) | 2.42 ( 0.75 ) |
| Jugular vein thrombosis | Vascular disorders | 3 | 14.47 ( 4.66 - 44.94 ) | 14.47 ( 37.52 ) | 14.43 ( 5.59 ) | 3.85 ( 2.18 ) |
| Oesophageal candidiasis | Infections and infestations | 3 | 6.76 ( 2.18 - 20.98 ) | 6.76 ( 14.7 ) | 6.75 ( 2.62 ) | 2.76 ( 1.09 ) |
| Cancer pain | Neoplasms benign, malignant and unspecified (incl cysts and polyps) | 3 | 9.25 ( 2.98 - 28.71 ) | 9.24 ( 22.03 ) | 9.23 ( 3.58 ) | 3.21 ( 1.54 ) |
| Tumour necrosis | Neoplasms benign, malignant and unspecified (incl cysts and polyps) | 3 | 24.29 ( 7.81 - 75.49 ) | 24.28 ( 66.7 ) | 24.19 ( 9.37 ) | 4.6 ( 2.93 ) |
